# Prenatal ultrasound diagnosis and prognosis assessment of fetal neck masses

**DOI:** 10.3389/fped.2025.1516356

**Published:** 2025-08-14

**Authors:** Peipei Zhang, Xining Wu, Yunshu Ouyang, Tianrui Yang, Yixiu Zhang, Hui You, Yan Lv, Yulin Jiang, Qing Dai, Hua Meng

**Affiliations:** ^1^ Department of Ultrasound, Peking Union Medical College Hospital, Chinese Academy of Medical Sciences and Peking Union Medical College, Beijing, China; ^2^ Department of Radiology, Peking Union Medical College Hospital, Chinese Academy of Medical Sciences and Peking Union Medical College, Beijing, China; ^3^ Department of Obstetrics and Gynecology, Peking Union Medical College Hospital, Chinese Academy of Medical Sciences and Peking Union Medical College, Beijing, China

**Keywords:** neck mass, prenatal ultrasound, magnetic resonance imaging, diagnosis, multidisciplinary treatment

## Abstract

**Objective:**

This study retrospectively analyzed the prenatal ultrasound features and outcomes of fetal neck masses to improve the understanding of fetal neck masses and provide evidence for prenatal consultation, prognosis assessment, delivery mode selection, and clinical intervention.

**Methods:**

From January 2018 to November 2023, 18 patients who underwent routine prenatal ultrasonography in the ultrasound department of Peking Union Medical College Hospital or who were referred to our hospital for the diagnosis of a fetal neck mass were retrospectively identified. Their prenatal ultrasound characteristics and pregnancy outcomes were examined and follow-up was conducted.

**Results:**

There were 18 cases of fetal neck masses. The mean gestational age at which the fetal neck mass was first detected was 27 ± 6 weeks (range 17–38 weeks). There were seven (39%) male fetuses, nine (50%) female fetuses, and two (11%) fetuses of undetermined sex. The clinical diagnosis was lymphangioma in 14 cases (78%), hemangioma in two (11%), teratoma in one (6%), and congenital goiter in one (6%). The maximum diameter of the fetal neck mass at the first ultrasound examination was 1.8–8.6 cm, and the median diameter was 4.3 (2.5, 6.5) cm. The median mass volume was 17.0 (3.5, 59.0) cm^3^ (range 1.0–219.0 cm^3^). The neck mass was cystic in nine cases, a cystic solid mass with compartmentalization in five cases, and a solid mass with a blood flow signal in four cases. Sixteen fetuses were delivered by elective cesarean section, while two were born via induced labor. The average postnatal follow-up time was 27 months, and the longest follow-up was 6 years. There were 13 cases (72%) with a favorable outcome and five (28%) with an unfavorable outcome.

**Conclusion:**

A fetal neck mass is a rare benign lesion. Accurate evaluation of the size and location of the cervical mass by prenatal ultrasound, auxiliary examinations such as magnetic resonance imaging, and assessments of clinical manifestations and related complications are crucial for appropriate prenatal consultation, prognosis assessment, delivery mode selection, and postpartum management. Multidisciplinary treatment is essential for the successful management of fetal cervical masses.

## Introduction

Fetal neck masses are rare malformations that can be located in the anterior, lateral, and posterior parts of the neck. Fetal neck masses are mostly benign but can compress the cervical airway and esophagus, posing potential risks such as preterm birth, airway obstruction, and intrauterine fetal death. Some cases require invasive uterine surgery, cesarean section, and ex utero intrapartum treatment (EXIT). Additionally, surgical intervention and continuous follow-up may be required after birth. Therefore, an accurate assessment of the fetal neck mass is important for appropriate prenatal consultation and management.

Prenatal ultrasound is the preferred method of screening for fetal abnormalities, including head and neck masses. Ultrasound can accurately assess the size, location, internal blood supply, and growth changes of the cervical mass, and can also comprehensively evaluate the pressure of the mass on neighboring structures and the amniotic fluid volume; these findings can help in the clinical identification of pregnant women who need close follow-up or emergency intervention, and enable the implementation of appropriate pregnancy care and perinatal management. This study retrospectively evaluated the prenatal ultrasound features and pregnancy outcomes of fetal neck masses, analyzed the prenatal factors affecting fetal airway obstruction and adverse outcomes, and provides evidence for prenatal consultation, prognosis assessment, and clinical intervention (1).

## Materials and methods

### Study population

This retrospective cohort study was conducted at Peking Union Medical College Hospital and included cases in which fetal neck masses were diagnosed either through routine prenatal ultrasound examination or referrals to our hospital. The study was approved by the Research Ethics Committee of Peking Union Medical College Hospital. The inclusion criteria were: (1) first detection of the fetal neck mass at 16–40 weeks gestation; (2) a space-occupying mass in the neck of the fetus that protruded from the skin surface and had a maximum diameter of more than 1 cm. The exclusion criteria were: (1) first detection of the fetal neck mass at a gestational age of less than 16 weeks; (2) nuchal fold thickening plus a few small cysts that did not protrude from the skin surface; (3) chromosomal abnormalities.

### Ultrasound examination

Ultrasound examinations were performed using color Doppler ultrasound diagnostic instruments (GE Voluson E10/E8, GE Healthcare; and Philips EPIQ7/IU22, Philips Healthcare) equipped with a transabdominal two-dimensional or three-dimensional convex array probe with frequency ranges of 2.0–5.0 MHz and 1.0–5.0 MHz, respectively. A systematic ultrasound examination was conducted to assess the general fetal growth and development, presence of congenital malformations, and condition of fetal appendages. When a fetal neck mass was found, it was evaluated in the coronal, sagittal, and transverse planes. The location and size of the mass were evaluated, and the volume was calculated as *π*/6 × *L* × *W* × *H*. The morphology, internal echogenicity, and blood flow within the mass were observed. The position of fetal neck mass and the relationship between the mass and trachea/esophagus were assessed. The fetus was also examined to detect the presence of a stomach bubble and polyhydramnios to assess whether there was esophageal compression; the severity was determined by the occurrence of complications such as fetal edema and heart failure. Fetal magnetic resonance imaging (MRI) was performed if necessary (MAGNETOM Avanto 1.5T: Simens Medical Solutions, Malvern, PA, USA).

### Follow-up

After the neck mass was detected, ultrasound examination was performed every 3–4 weeks to observe the mass size, growth rate, relationship with the trachea, and fetal complications. When fetal distress or serious complications occurred, a multidisciplinary fetal treatment team (comprising experts in the fields of obstetrics, ultrasound, radiology, neonatology, pediatric surgery, and anesthesiology) discussed the timing and methods of potential pregnancy termination and neonatal care. If fetal imaging determined that there was a high likelihood of severe airway obstruction (due to airway obstruction or excessive amniotic fluid), then EXIT was considered. After birth, neonates were referred to Capital Center for Children's Health, a tertiary pediatric hospital, for further management, including surgical intervention when clinically indicated. Pathological examinations of relevant specimens were conducted at this center, with the results serving as the basis for final diagnosis.

The medical records were reviewed and telephone follow-up was conducted postnatally to document the gestational age at delivery, mode of delivery, infant's birth weight, general growth status, surgical treatments, and pathological examination results. Follow-up was maintained for a minimum of 6 months postnatally, with some cases monitored until the infants reached 6 years of age. A favorable outcome was defined as live-born infants who, after surgery or other interventions, showed no significant developmental differences from other infants, while an unfavorable outcome included induced labor and neonatal death.

### Statistical analysis

SPSS 20.0 software was used for statistical analysis. The measurement data conforming to normal distribution are expressed as mean ± standard deviation, while the measurement data not conforming to normal distribution are expressed as median (quartile). The comparison of quantitative parameters between the two groups was performed using the Mann–Whitney *U* non-parametric test, while the comparison of categorical variables was performed using the Chi-squared test and Fisher's exact probability method. *P* < 0.05 was considered statistically significant.

## Results

### General situation

Eighteen patients met the study eligibility criteria. The mean age of the pregnant women was 32 ± 5 years (range 25–43 years), and the mean gestational week in which the fetal neck mass was first detected was 27 ± 6 weeks (range 17–38 weeks). The clinical diagnosis was lymphangioma in 14 cases (78%), hemangioma in two (11%), teratoma in one (6%), and congenital thyroid goiter in one (6%), as summarized in Table 1.

**Table 1:** Ultrasonic characteristics and pregnancy outcome of patients with fetal neck masses.

ID	Age(year)	GA at diagnosis (w)	Position of Neck	Maximum Diameter(cm)	Volume (cm^3^)	Property	Airway compression	Malformations	GA at birth (w)	Delivery mode	EXIT	Newborn sex	Diagnosis	Clinical Follow-up	Follow-up time
1	25	22 + 2	Leteral	1.8	1.0	Cystic			37	Cesarean section	—	M	Lymphangioma	Disappeared before birth	5 Years
2	29	17 + 0	Dorsal	2.4	1.9	Cystic		Few pleural effusion	38	Cesarean section	—	F	Lymphangioma	Disappeared before birth	3 Years
3	26	27 + 2	Dorsal	1.8	2.2	Cystic			38	Cesarean section	—	M	Lymphangioma	Disappeared before birth	5 Years
4	36	23 + 2	Lateral	2.3	3.7	Cystic			37	Cesarean section	—	M	Lymphangioma	Disappeared before birth	4 Years
5	35	23 + 6	Dorsal	3.0	5.3	Cystic			38	Cesarean section	—	M	Lymphangioma	Disappeared after birth	6 Years
6	33	33 + 3	Lateral	3.9	6.9	Solid cystic			38	Cesarean section	—	F	Lymphangioma	Disappeared after birth	1 Year
7	33	36 + 3	Lateral	4.6	25.7	Solid cystic			39	Cesarean section	—	M	Lymphangioma	Disappeared after birth	3 Years
8	32	37	Dorsal	6.4	64.1	Cystic			38	Cesarean section	—	F	Lymphangioma	Interventional treatment	6 Years
9	43	26 + 2	Lateral	7.5	189.0	Solid cystic	Yes	Polyhydramnios	36	Cesarean section	Yes	F	Lymphangioma	Resection 46 days after birth	1 Year
10	35	25 + 2	Lateral	5.0	45.8	Cystic			37	Cesarean section	Yes	F	Lymphangioma	Resection 10 days after birth	1 Year
11	33	37 + 4	Anterior	6.4	44.7	Solid		Pleural effusion, Polyhydramnios	39	Cesarean section	—	M	Lymphangioma	Neonatal death	1 Month
12	38	30	Dorsal	2.5	3.3	Cystic			36	Cesarean section	—	F	Lymphangioma	Neonatal death	1 Day
13	27	20 + 4	Anterior	7.6	159.0	Solid cystic		Abnormal hands	21	Induction of Labour	—	—	Lymphangioma	Death	
14	26	17 + 0	Dorsal	3.5	7.6	Cystic		Edema, cardiac malformation	17	Induction of Labour	—	—	Lymphangioma	Death	
15	28	34 + 4	Lateral	6.5	53.8	Solid			38	Cesarean section	—	M	Hemangioma	Resection 33 days after birth	1 Year
16	33	28	Anterior	5.1	49.3	Solid		Polyhydramnios	37	Cesarean section	Yes	F	Hemangioma	Resection 30 days after birth	6 Months
17	29	23 + 5	Anterior	8.6	219.0	Solid cystic	Yes	Edema, Pleural effusion, Polyhydramnios	31	Cesarean section	Yes	M	Immature teratoma	Neonatal death	1 Day
18	32	30	Anterior	5	50.4	Solid		Polyhydramnios	38	Cesarean section	Yes	F	Thyroid goiter	Medical treatment	6 Months

GA, gestational age; EXIT, Ex utero intrapartum treatment; M, male; F, female.

### Imaging examination of the neck mass

Among the 18 fetal neck masses, six were located in the anterior neck, seven were located in the lateral neck, and five were located in the posterior neck. There was significant variation in the size of the fetal neck masses; the maximum diameter on initial ultrasound detection ranged from 1.8 to 8.6 cm, with a median of 4.3 (2.5, 6.5) cm. The mass volume ranged from 1 to 219 cm^3^, with a median of 17 (3.5, 59) cm^3^. Nine cases showed a cystic mass, five showed a cystic solid mass with compartmentalization, and four showed a solid mass with an internal blood flow signal. Two cases (cases 9 and 17) showed obvious posterior or lateral curvature of the fetal head, airway stenosis, and deviation due to the large mass volume. Pleural effusion was present in three cases (cases 2, 11, and 17), while two had fetal edema (cases 14 and 17). Polyhydramnios was observed in five cases (cases 9, 11, and 16–18). In one case, the fetus had complex cardiac malformations and pulmonary artery atresia. One fetus (case 13) had abnormal posture of both hands. Seven patients underwent MRI examination at a gestational age of 28–26 weeks. Two cases (cases 9 and 17) had fetal esophagus-airway compression and displacement, which was consistent with the ultrasonographic findings. In the other five cases (case 10–11, 15–16, and18), there were no findings of fetal esophago-airway compression displacement.

### Fetal outcomes

Sixteen patients opted for elective cesarean section and delivered seven (39%) male neonates and nine (50%) female neonates. Two patients (11%) chose to have their labor induced due to their fetus having a cardiac malformation or larger lesion size, and the sex of the neonates was not recorded. The average follow-up of the 16 patients was 27 months, and the longest follow-up was 6 years after birth. There were 13 fetuses (72%) with favorable outcomes and five (28%) with unfavorable outcomes.

The fetal neck masses disappeared spontaneously in seven cases (cases 1–7), all of which were lymphangiomas, with four located on the lateral aspect of the neck and three located in the posterior region. Five fetuses had cystic masses, and two had cystic solid masses. The fetal neck mass disappeared before birth in four cases, and disappeared 3–6 months after birth in three cases. In the cases with spontaneous mass disappearance, the mean maximum diameter of the neck mass was 2.8 ± 1.1 cm (range 1.8–4.6 cm) and the median volume was 3.7 (1.9, 6.9) cm^3^ (range 1.0–25.7 cm^3^). Among the neck masses that failed to disappear spontaneously, the mean maximum diameter was 5.8 ± 1.9 cm (range 2.5–8.6 cm) and the median mass volume was 50.4 (44.7, 159.0) cm^3^ (range 3.3–219.0 cm^3^). Compared with the group without mass disappearance, the group with spontaneous mass disappearance had a significantly smaller maximum diameter (*P* = 0.001) and mass volume (*P* = 0.002). In the group with spontaneous mass disappearance, all cases had a maximum lesion diameter of <5 cm.

**Figure 1:**
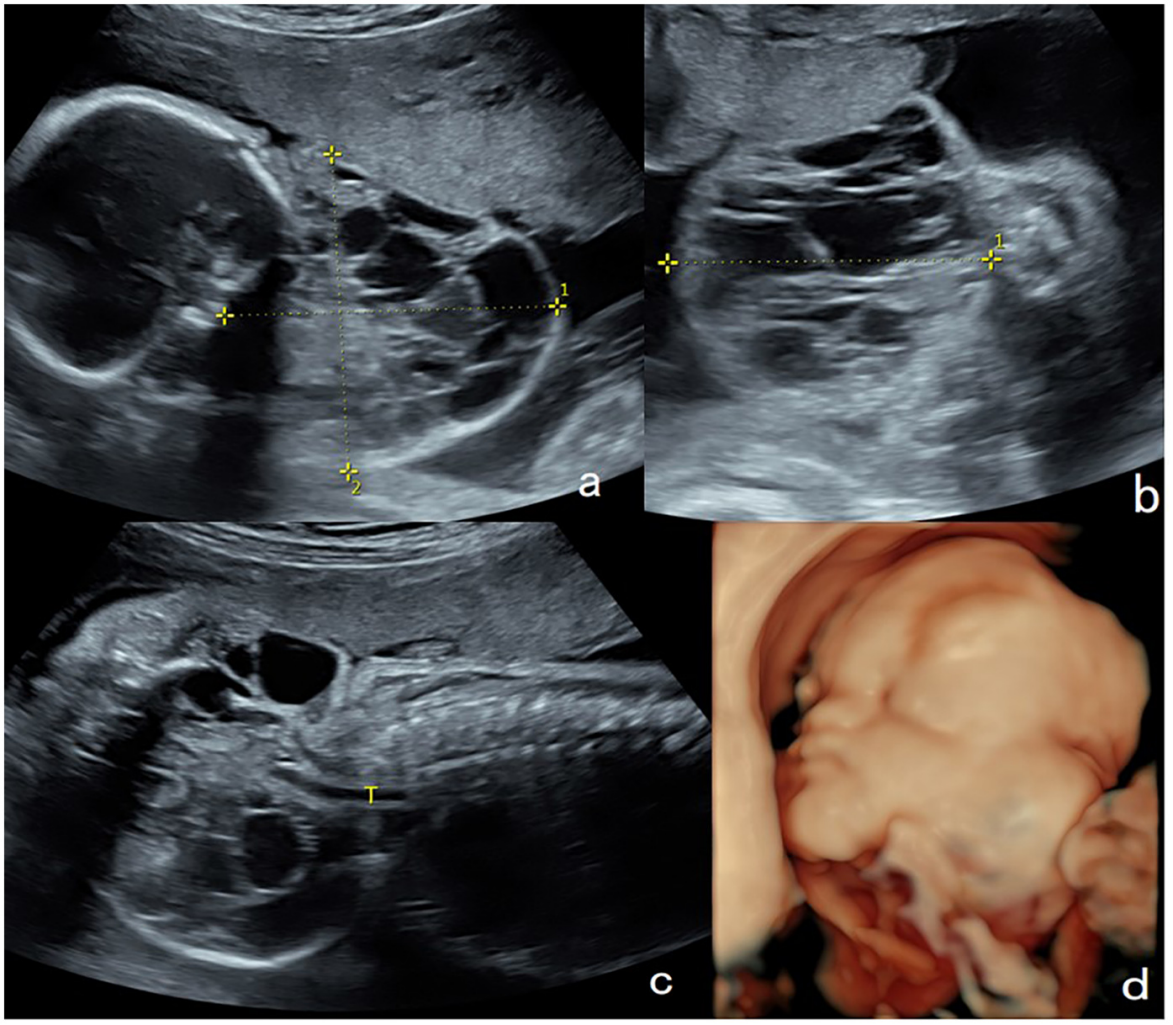
Lymphangioma in the neck of a fetus (case 9). A solid cystic mass (7.5 cm × 6.5 cm × 7.1 cm) with internal separations was found in the lateral neck of the fetus at 26 weeks of gestation. **(a,b)** On color Doppler flow imaging, the blood flow signals in the mass were observed in the spot strips. **(c)** The lower tracheal segment was visible, while the upper tracheal segment was not satisfactorily visualized. **(d)** Three-dimensional ultrasound showed that the mass was mainly located on the lateral aspect of the neck. The mass was 10.9 cm × 10.4 cm × 8.5 cm at 36 weeks of gestation. Elective cesarean section was performed at 36 weeks + 4 days of gestation, and tracheal intubation was successful during ex utero intrapartum treatment. The mass was surgically removed 46 days after birth and was pathologically diagnosed as a lymphangioma with a favorable outcome.

**Figure 2:**
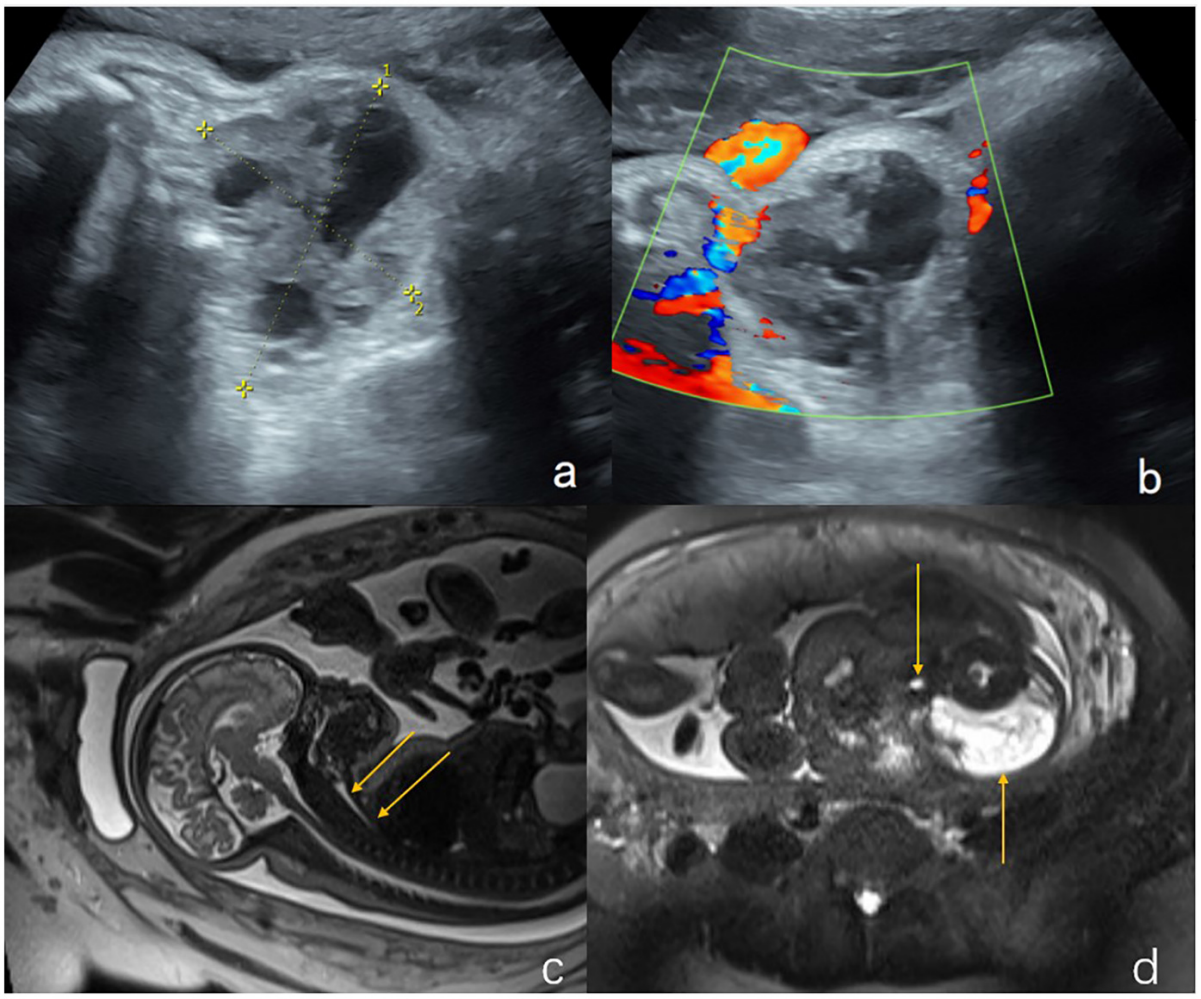
Lymphangioma in the neck of a fetus (case 10). **(a,b)** Sagittal and axial views of the cervical mass by two-dimensional ultrasonography. A mass on the lateral aspect of the fetal neck (5 cm × 4.4 cm × 4 cm) was found at 25 weeks of gestation. **(c,d)** Sagittal and axial T2-weighted showed a lobulated heterogenous mass at left neck of the fetus which is hyperintense with hypointense separation within it. Magnetic resonance imaging showed that the left neck of the fetus had a lumpy-long T2 signal that was not uniform and had low signal separation within it. The maximum cross-section of the sagittal position was 5.8 cm × 4.0 cm, the pharyngeal cavity structure was visible, and the trachea was visible throughout, without obvious stenosis. The cervical mass was 6.7 cm × 5.7 cm × 3.1 cm in the 37th week of gestation. Elective cesarean section was performed at 37 weeks + 3 days of gestation. Tracheal intubation was successful in ex utero intrapartum treatment during delivery. After the operation, the newborn had a favorable outcome and is currently 1 year old with normal height, weight, and intelligence.

**Figure 3:**
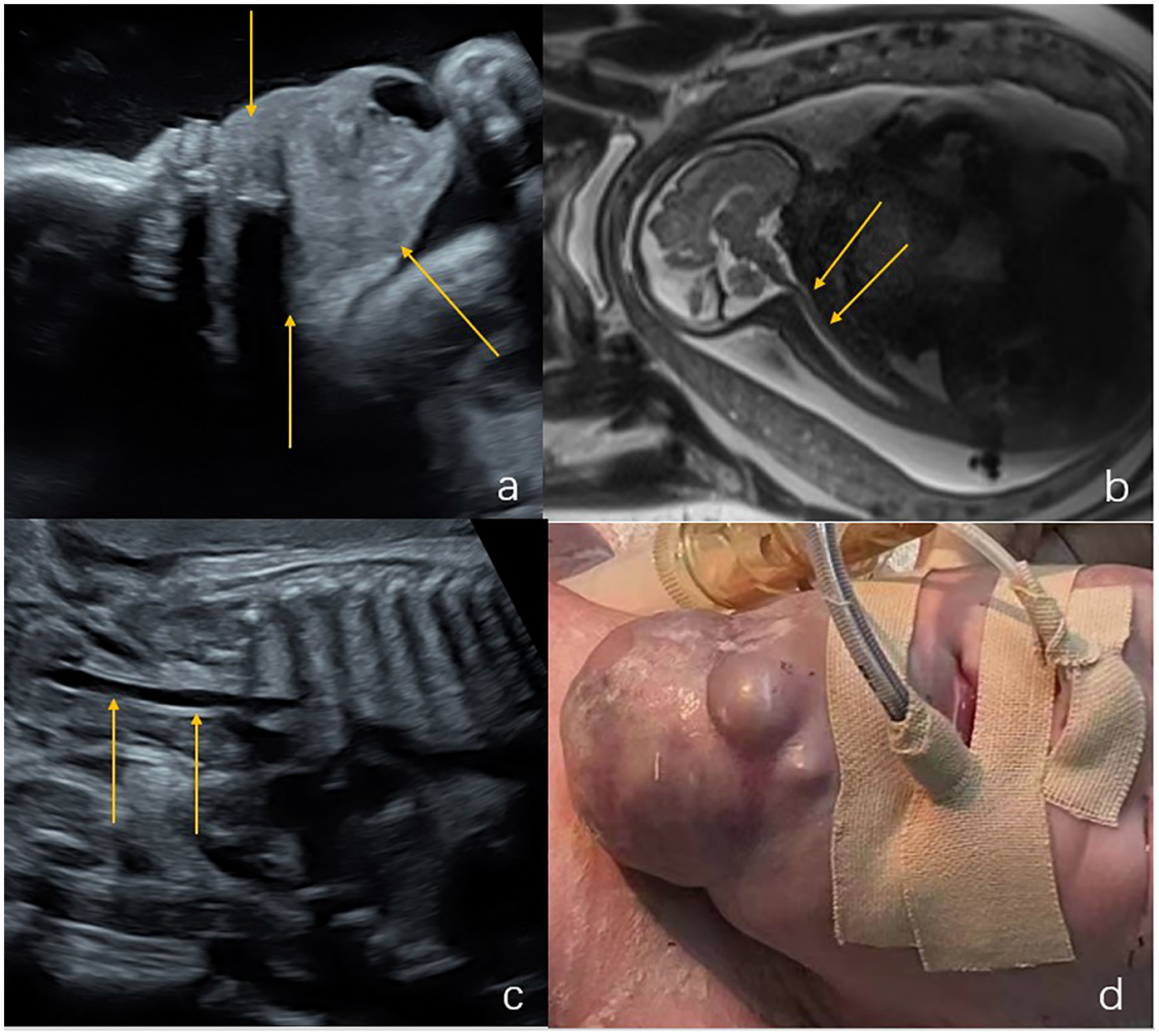
Hemangioma in the neck of a fetus (case 16). **(a)** Ultrasound examination at 28 weeks of gestation revealed a solid mass (6.9 cm × 5.5 cm × 5 cm) in the submandibular region and neck (indicated by the arrows). **(b)** Sagittal T2-weighted magnetic resonance imaging, the structure of the entire trachea was visible (indicated by the arrows). **(c)** Ultrasound examination at 37 weeks of gestation showed that the mass was 7.2 cm × 6.0 cm × 5.9 cm. The trachea was visible (indicated by the arrow), and an elective cesarean section was performed at 37 weeks + 3 days of gestation. **(d)** The tracheal intubation was successful in ex utero intrapartum treatment during delivery. The neck mass of the newborn is shown by the arrow. The mass was surgically removed 30 days after birth and was pathologically diagnosed as a hemangioma with a favorable outcome.

Four neonates (22%) underwent surgical excision of the neck mass 10 days–2 months after birth and had a favorable outcome after the operation. The postoperative pathologic findings were lymphangioma in two cases (Figure 1-case 9, Figure 2-case 10) and hemangioma in two (case 15, Figure 3-case 16). In one case (case 8) in which the clinical diagnosis was a lymphangioma with a maximum diameter of 6.4 cm measured by ultrasound, the mass was absorbed after a sclerosing agent was injected into the neck mass. In one case (case 18), a solid mass was found in the anterior neck during pregnancy, with a maximum diameter of 5.0 cm, uniform echo, and abundant internal blood flow signal, which was considered to be fetal thyroid goiter. Umbilical cord blood aspiration indicated fetal hypothyroidism, and family whole exome sequencing indicated TG recheck heterozygous gene variation. After birth, the newborn continued to take oral levothyroxine sodium; the thyroid function gradually improved and ultrasonography showed that the thyroid volume returned to normal.

**Figure 4:**
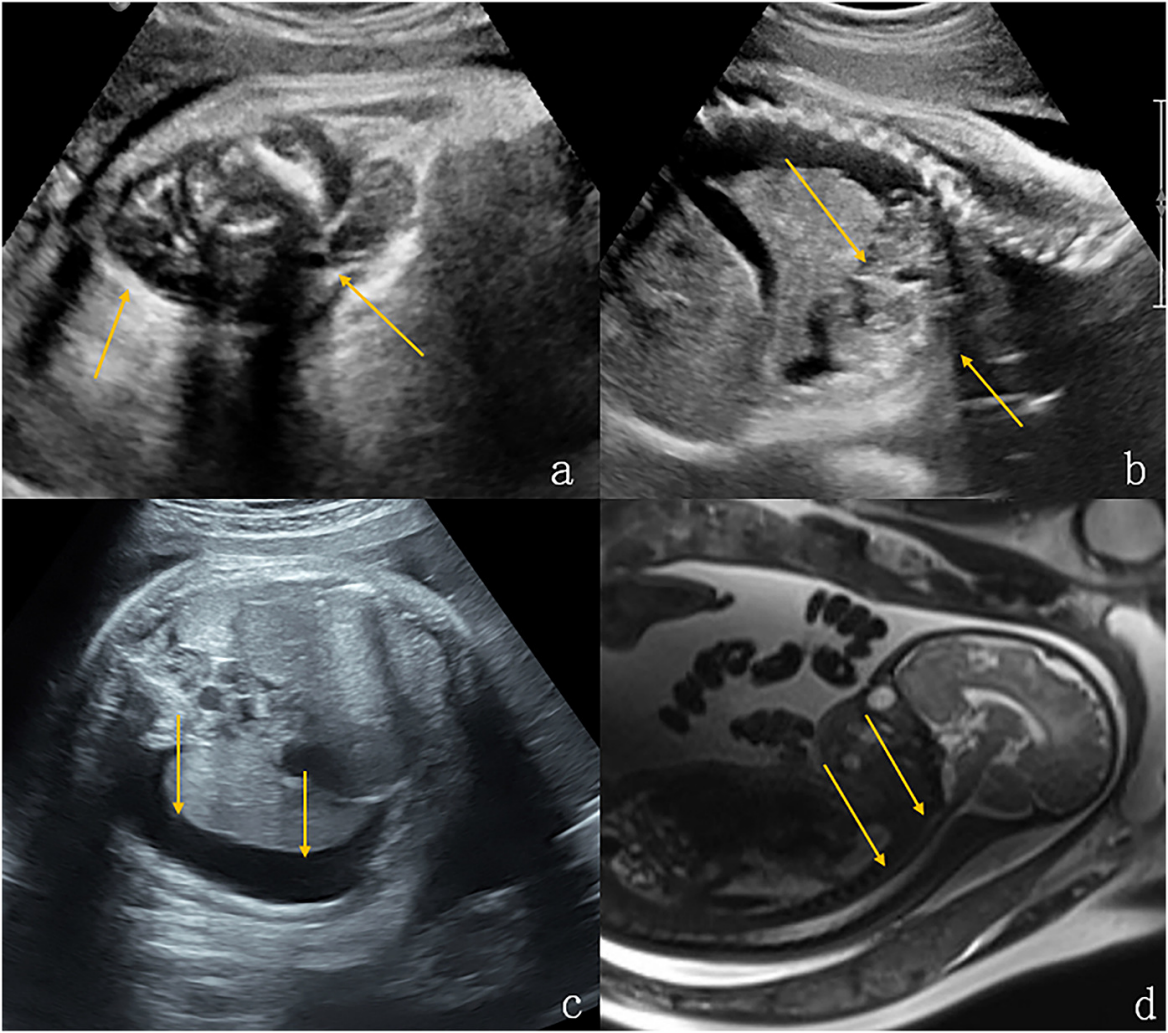
Lymphangioma in the neck and upper mediastinum of a fetus (case 11). **(a,b)** Solid cystic masses were found in the neck and upper mediastinum of the fetus at 37 weeks gestation (indicated by the arrows). **(c)** There was concomitant bilateral pleural effusion (shown by the arrows). **(d)** Sagittal T2-weighted magnetic resonance imaging showed that the structure of the entire trachea was visible (shown by the arrows). The newborn had dyspnea after being delivered via cesarean section at 39 weeks of gestation. After conservative treatment, the size of the tumor increased and the pleural drainage effusion increased. The infant died 45 days after birth.

**Figure 5:**
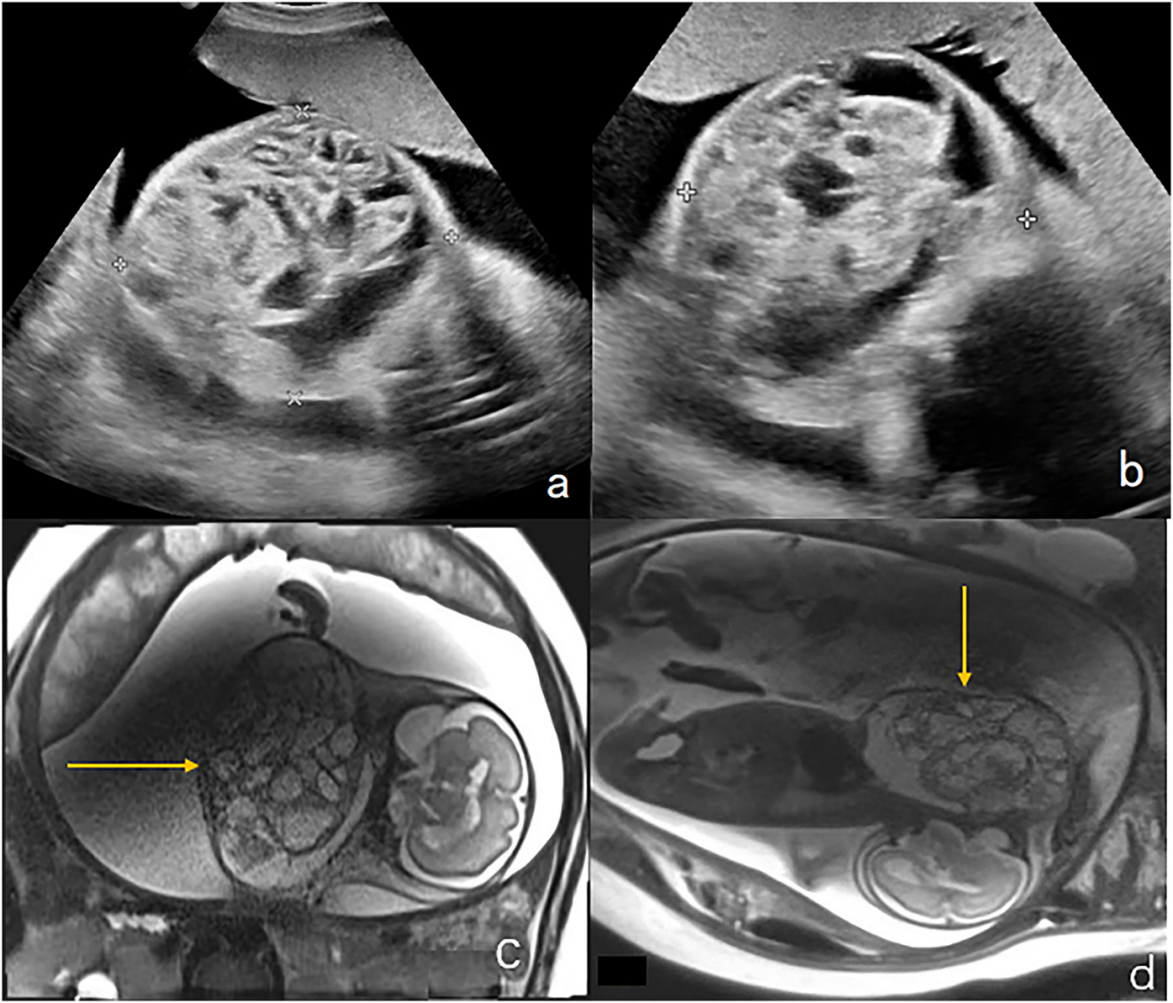
Immature teratoma in the anterior neck of a fetus (case 17). **(a,b)** Ultrasound examination at 23 weeks of gestation revealed a mass in the front of the neck measuring 8.6 × 7.2 × 6.8 cm, with a maximum amniotic fluid depth of 7 cm. **(c)** Coronal T2-weighted magnetic resonance imaging showed an irregular solid cystic mass in the front neck of the fetus, with multiple compartments, long T2 signals, and solid T1 and T2 soft tissue components(shown by the arrows). **(d)** Sagittal T2-weighted magnetic resonance imaging, the fetal head was substantially tilted posteriorly. The mass and amniotic fluid increased gradually over the following weeks. The maximum mass diameter and amniotic fluid depth were 14 cm and 13 cm, respectively, at 31 weeks of gestation. An emergency cesarean section was performed at 31 weeks + 5 days of gestation due to premature membrane rupture. Intubation during ex utero intrapartum treatment failed and the newborn died of asphyxia. The postoperative pathology showed that the mass was an immature teratoma of the neck.

Two cases underwent induction of labor during the second trimester due to a larger lesion size (case 13) and edema with cardiac malformation (case 14). Three neonates died after birth (cases 11, 12, and 17). One neonate with lymphangioma (case 11-Figure 4) had solid cystic masses in the neck and upper mediastinum at 37 weeks of gestation. The lesions were extensive and were combined with bilateral pleural effusion and lateral ventricular widening. The newborn had dyspnea after being delivered via cesarean section at 39 weeks of gestation, and was intubated and received ventilator assistance. The neck masses were treated conservatively with closed thoracic drainage and bleomycin injection sclerotic treatment. However, the masses enlarged and the pleural effusion increased after treatment, and the infant died 45 days after birth. One fetus with lymphangioma (case 12) was found to have a cystic mass in the neck and back (maximum diameter 2.5 cm; volume 3.3 cm) at 30 weeks of gestation, with no other structural malformations. Ultrasound examination performed 5 weeks later showed no substantial enlargement of the mass. After being delivered via cesarean section at 36 weeks, the newborn had low muscle tone, abdominal distension, pleural effusion, abdominal effusion, and poor score. The newborn died 4 h after intubation. In one fetus pathologically diagnosed as having an immature cervical teratoma (case 17-Figure 5), ultrasound examination at 23 weeks of gestation revealed an anterior cervical mass with a maximum diameter of 8.6 cm and a maximum amniotic fluid depth of 7 cm. The mass and amniotic fluid increased gradually to a maximum mass diameter of 14 cm and the amniotic fluid depth of 13 cm at 31 weeks of gestation. An emergency cesarean section was performed at 31 weeks + 5 days of gestation due to premature membrane rupture. Endotracheal intubation during EXIT failed and the newborn died of asphyxia.

### EXIT

Five patients (cases 9, 10, and 16–18) underwent elective cesarean section and EXIT endotracheal intubation after multidepartment consultation. Of these, two neonates (cases 9 and 17) had signs of airway compression before delivery, and four had polyhydramnios before delivery. The mean gestational age at delivery for the five patients was 36 weeks (range 31–38 weeks), and the size of the cervical mass ranged from 5.0 cm × 4.4 cm × 4.0 cm to 18.5 cm × 13.0 cm × 8.0 cm. After endotracheal intubation, four neonates were successfully treated and had a favorable outcome. One neonate with an immature teratoma of the anterior neck (case 17) experienced EXIT tracheal intubation failure and died due to the large volume of the anterior cervical mass and poor airway conditions.

## Discussion

Fetal neck masses are usually benign lesions, such as lymphangioma, hemangioma, teratoma, thyroid goiter, and laryngeal cyst. However, rare malignant tumors have also been reported, such as malignant melanoma, neuroblastoma, and malignant rhabdoid tumor (2–5). The fetal neck mass may be cystic or solid. Prenatal ultrasound can help identify the nature of the neck mass based on its location, size, type, and blood flow. The most common lymphangioma in the posterior triangle is a cystic, multilocular, or solid mass of varying sizes due to dysplasia of the lymphatic system, with no internal blood flow signal (6–8). In the anterior triangle, teratoma, hemangioma, and thyroid goiter are most common. Congenital teratoma is a form of germ cell tumor that originates from the body's midline and is composed of three germ layers with complex components. Morphologic heterogeneity reflects the complex composition of teratomas. Benign mature teratoma lesions are mostly cystic, whereas immature lesions tend to be more solid. Most teratomas have a combination of cystic and solid components, accompanied by calcifications. Hemangiomas form solid or cystic structures from proliferating vascular endothelial cells, with colored blood flow signals and calcification within the mass. Calcification has always been considered a very typical sign of teratoma; however, hemangiomas with venous components may have a similar appearance because they often contain calcified phleboliths and therefore may be misdiagnosed as teratomas. A thyroid goiter is a solid mass of uniform texture, without separation, often located in the center line, with left and right symmetry. Malignant masses of the neck are usually solid tumors that progress rapidly (9–11).

Prenatal ultrasound can identify fetuses at risk by evaluating the size of the cervical mass (12). In the present study, the maximum diameter of self-resolving neck masses was less than 5 cm. However, when the mass is larger than 5 cm, it may lead to corresponding indirect signs such as fetal difficulty in closing the mouth; neck bending or excessive extension to the opposite side; compression of the trachea and esophagus, affecting fetal swallowing of amniotic fluid and resulting in polyhydramnios; or compression of neck and facial structures leading to deformity of facial structures, mandibular deformities, poor development of neck muscle tissue, and cranial nerve dysfunction (13, 14). Yu et al. (15) suggested that tumors with a diameter exceeding 50 mm may cause difficult labor and necessitate consideration of cesarean section for delivery.

It is crucial to evaluate the location, characteristics, and associated complications of fetal neck masses for prognostic assessment and selection of the delivery mode. The three-dimensional ultrasonic surface imaging model can show the attachment site of the mass and the relationship between the mass and the surrounding tissue. Lymphangiomas and hemangiomas are usually located in the posterior or posterolateral regions of the neck, and often cause displacement of the trachea or esophagus. The outcome of fetuses with lymphangiomas and hemangiomas in the neck is relatively favorable. In the present study, 10 of the 11 fetuses (91%) with lymphangioma had a favorable outcome. However, when the mass is located in the anterior region of the neck, it easily compresses the trachea and esophagus, resulting in fetal airway obstruction, impaired swallowing, excessive amniotic fluid, and premature delivery, potentially resulting in neonatal hypoxia and death during delivery. Teratomas of the neck are mostly located in the anterior region and carry a higher risk of airway obstruction than lymphangiomas and hemangiomas (16–21). Furthermore, an anterior neck mass easily expands into the mediastinum, which is an additional risk factor for airway obstruction (22, 23). When the fetal neck mass is isolated, the outcome is relatively favorable; however, when the mass is associated with other abnormalities such as heart malformation, excessive amniotic fluid, fetal skin edema, pleural effusion, and abdominal effusion, the outcome is unfavorable (24–27). In the present study, there were two fetuses with pleural effusion and increased amniotic fluid, both of whom had an unfavorable outcome. Three fetuses only had concomitant polyhydramnios, and thus their outcome was favorable. One fetus had concomitant heart malformation and pulmonary atresia, and the pregnancy was terminated owing to the unfavorable outcome.

A fetal neck mass may cause partial or complete airway obstruction. Prenatal ultrasound enables direct visualization of the larynx and trachea to detect any signs of compression. The fetal tracheal diameter can also be measured and the Z-score calculated to quantitatively assess the degree of tracheal compression (28). Moreover, ultrasonography can indirectly assess esophageal obstruction by identifying the absence of a visible stomach bubble, decreased swallowing, tongue protrusion, and polyhydramnios. Ultrasound is the preferred method of follow-up observation and can detect changes in mass size and amniotic fluid volume in time, which can help identify those situations that require emergency termination of pregnancy (16). Fetal MRI is an effective complement to ultrasound that can further evaluate fetal neck masses to give the maximum vertical diameter, tracheal esophageal displacement index, and impact of the mass on the trachea to predict airway difficulties and mortality (29). Generally, fetal MRI should be considered in every fetus with neck masses. MRI helps assess the severity, cause, and anatomical details of airway compromise (30). Especially when the mass is large, the boundary between the mass and the surrounding tissue is not clear, and the source and scope of the ultrasonic evaluation of the mass are not clear, further MRI examination is recommended. At the same time, MRI can provide more diagnostic information when ultrasound images show uneven echoes, suggesting that the neck mass may have complex tissue composition or a risk of malignancy. MRI is not affected by fetal position, oligohydramnios, bone overlay, or maternal obesity (3, 9, 31). Fetal neck masses that carry a risk of partial or complete airway obstruction may necessitate an EXIT procedure. The EXIT procedure allows for partial fetal delivery with continued maintenance of uteroplacental perfusion and gas exchange while measures are taken to secure the fetal airway prior to completion of the delivery. When the neck mass obstructs the airway, EXIT improves the prognosis and reduces fetal mortality from 10% to 57% to less than 8% (12, 32, 33). In the present case series, five fetuses underwent EXIT; four survived after tracheal intubation with a favorable outcome, and one was aborted due to airway stenosis. EXIT is a complex multidisciplinary treatment process that requires cooperation among the departments of Fetal Imaging, Obstetrics, Neonatology, Pediatric Surgery, Anesthesiology, and others. An accurate assessment of the fetal airway and other conditions by prenatal ultrasound benefits pregnant women in the multidisciplinary diagnosis and treatment process.

This study has certain limitations. First, the relatively small sample size primarily stems from the rarity of fetal neck masses; future research could expand the cohort by integrating multi-source data or extending the observation period. Second, advanced imaging technologies such as augmented reality or virtual reality were not incorporated herein. We plan to explore the application of these emerging tools in future studies to further enhance the comprehensive visualization of fetal neck masses. Third, given the rarity of fetal neck masses and the strict indications for intrauterine treatment, this study did not include content related to in-utero intervention. Future research could further explore the feasibility and application value of intrauterine treatment with accumulated cases.

In summary, prenatal ultrasound can be used to found and evaluate fetal neck masses. An evaluation of the predicted pregnancy outcome requires assessments of the neck mass volume and the presence of other abnormalities. Prenatal ultrasound can accurately evaluate the size and position of the neck mass and dynamically monitor the fetal airway pressure and amniotic fluid flow. When combined with fetal MRI, the source of the mass, its internal composition and its influence on the surrounding tissue can be further determined, which is helpful for prenatal consultation and selection of the delivery mode and provides a basis for prognosis assessment and postpartum management. Multidisciplinary treatment is essential for the successful management of fetal neck masses.

## Data Availability

The original contributions presented in the study are included in the article/Supplementary Material, further inquiries can be directed to the corresponding authors.
